# Eviction notice served on *Toxoplasma*

**DOI:** 10.7554/eLife.76246

**Published:** 2022-01-25

**Authors:** Juan C Sánchez-Arcila, Kirk DC Jensen

**Affiliations:** 1 Department of Molecular and Cell Biology, University of California Merced Merced United States; 2 Health Science Research Institute, University of California, Merced Merced United States

**Keywords:** parasites, egress, cell death, interferon gamma, growth restriction, Toxoplasma, vacuole, Other

## Abstract

The gene *RARRES3* uses an unexpected strategy to eliminate the parasite *Toxoplasma gondii* from human cells.

**Related research article** Rinkenberger N, Abrams ME, Matta SK, Schoggins JW, Alto NM, Sibley LD. 2021. Overexpression screen of interferon-stimulated genes identifies RARRES3 as a restrictor of *Toxoplasma gondii* infection. eLife **10**:e73137. doi: 10.7554/eLife.73137

Cells have a variety of defense mechanisms for eliminating parasites, bacteria and other pathogens. To evade eviction, some of these pathogens sequester themselves inside structures called vacuoles once they are inside the cell. This allows the pathogens to grow ‘rent-free’, scavenging food from the cytosol without triggering the many ‘trip wires’ that lie immediately beyond the vacuole.

Many parasites rely on this strategy to survive, including *Toxoplasma gondii*, the microorganism that causes toxoplasmosis. When *T. gondii* is ingested by a human or other warm-blooded animal, the parasite invades cells lining the small intestine, using the plasma membrane of the cells to form the membrane of the vacuole (Figure 1; Suss-Toby et al., 1996). Once inside, the parasite starts to divide and mature into a new form that then gets released via a process called egress; the freshly egressed parasite then seeks out new cells to invade and quickly spreads throughout the body. *T. gondii* is considered one of the world’s most successful parasites because, once fully developed, it can infect virtually any cell with a nucleus. So, how does the host’s immune system remove this unauthorized occupant?

**Figure 1.**
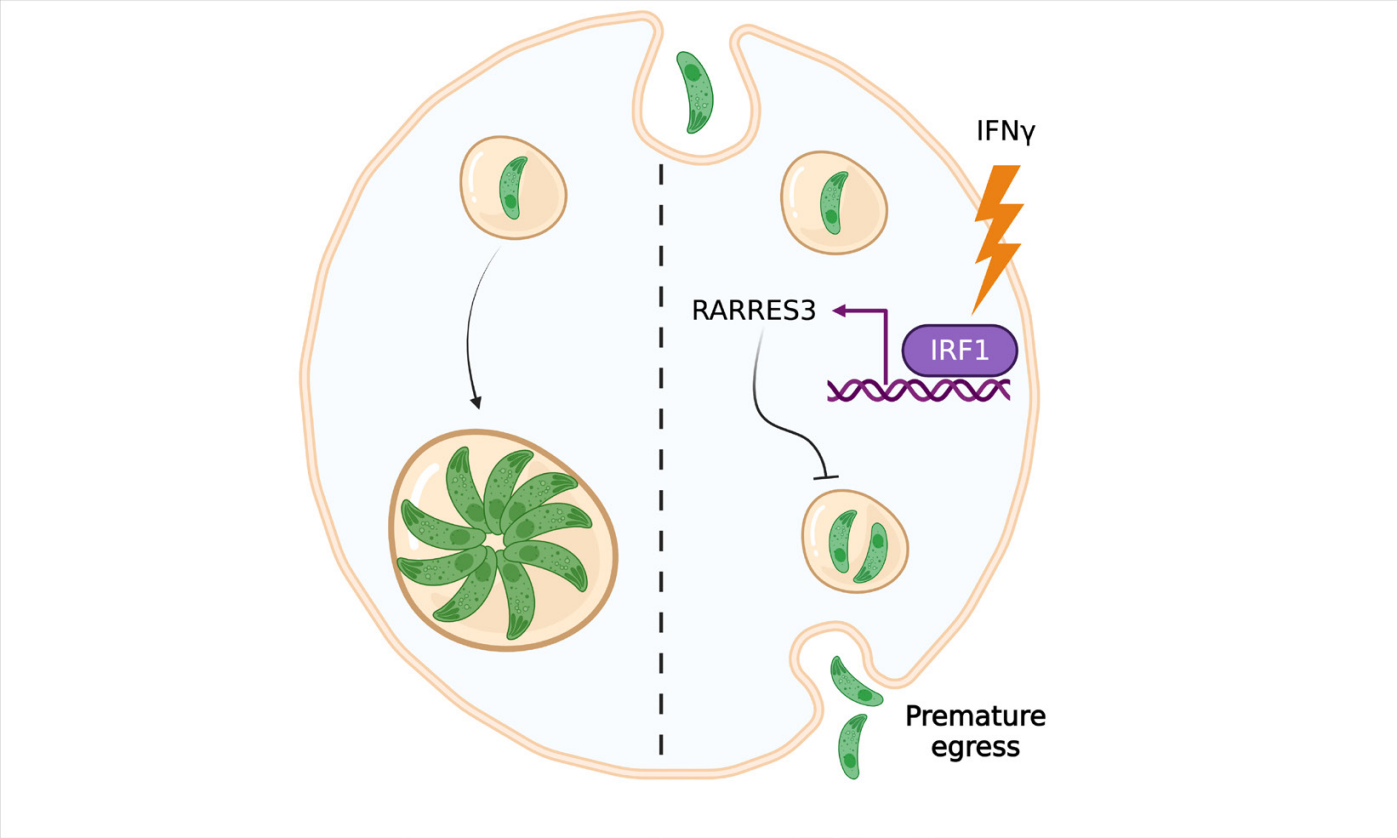
A new way of evicting ***Toxoplasma gondii*** from cells. In resting cells, *T. gondii* (green) creates a vacuole surrounded by a membrane, inside which it can replicate and grow without being destroyed by the immune system (left). However, when the immune system stimulates the cell with a protein called interferon gamma (IFNγ; right), multiple genes are activated, including a gene called *RARRES3* which codes for a phospholipase enzyme and is regulated by a transcription factor called IRF1. Rinkenberger et al. show that *RARRES3* restricts vacuolar growth and causes *T. gondii* to prematurely exit the cell.

Image credit: Figure created using BioRender.com.

Most of the immune responses against *T. gondii* are regulated by a protein messenger called interferon gamma (IFNγ), which causes infected cells to transcribe hundreds of genes coding for proteins that stop the parasite from replicating (Pfefferkorn et al., 1986; Suzuki et al., 1988; Schoggins, 2019). In mice, IFNγ activates two sets of genes: one set codes for immunity-related GTPases (IRGs), and the other codes for guanylate binding proteins (GBPs). These proteins surround and disrupt the vacuole membrane, thereby killing the parasite growing inside (Martens et al., 2005; Ling et al., 2006; Yamamoto et al., 2012).

It is well established that the level of damage caused by different strains of *T. gondii* depends on their capacity to deactivate IRGs (Hunter and Sibley, 2012). Humans, however, do not have this IRG system, and much less is known about how our bodies kill off *T. gondii* (Bekpen et al., 2005; Saeij and Frickel, 2017). Now, in eLife, David Sibley and colleagues from Washington University and the University of Texas Southwestern Medical Center – including Nicholas Rinkenberger as first author – report how an IFNγ-stimulated gene called *RARRES3* restricts *T. gondii* infections in human cells (Rinkenberger et al., 2021).

First, the team used a forward genetic approach that involved individually overexpressing hundreds of IFNγ-stimulated genes to see which ones interfered with the growth and replication of *T. gondii*. These experiments, which were carried out on human cells cultured in the laboratory, led to the discovery of *RARRES3*, a gene that codes for an understudied phospholipase enzyme that plays a role in lipid metabolism (Mardian et al., 2015).

Because the parasitic vacuole cannot fuse with other compartments, the infected cell cannot dispose of *T. gondii* by transporting it to the cell surface or degrading it in its lysosome (Mordue et al., 1999). Therefore, most IFNγ-stimulated genes eliminate the parasite by either disrupting the membrane surrounding the vacuole or ‘blowing up’ the infected cell (Saeij and Frickel, 2017). However, Rinkenberger et al. found that *RARRES3* does not trigger either of these defense mechanisms. Instead, it reduces the size of the vacuole, causing *T. gondii* to egress before it has fully matured (Figure 1). This mechanism was shown to be specific to *RARRES3*, as this effect was not observed when the activity of the enzyme encoded by the gene was inhibited. In addition, restriction of the parasite’s vacuole was found to work independently from all other pathways known to induce cell death.

So, how does the parasite receive the eviction notice served by the *RARRES3* gene, and how does the phospholipase enzyme encoded by this gene shrink the vacuole? *T. gondii* feeds on a variety of biomolecules and scavenges lipids from lipid droplets in the cytosol of its host cell (Nolan et al., 2017). Perhaps the enzyme starves the parasite by simply metabolizing these lipids before the parasite can get to them. Or maybe it somehow stops the parasite from using these lipids to expand the membrane around the vacuole. Interestingly, *RARRES3* was found to only restrict strains of *T. gondii* that do not cause severe disease in mice and possibly humans. This suggests that there are likely to be other unknown mechanisms that explain why some strains of *T. gondii* cause more dangerous effects than others.

At first glance, it may seem that removing *T. gondii* from the cell (without killing it) will actually help the parasite to spread; however, there are some advantages to this strategy. First, it exposes the parasite to the extracellular environment, where it will encounter other components of the immune system (Souza et al., 2021). Second, it is possible that restricting the parasite’s food intake means it cannot build all the machinery it needs to invade new cells before being prematurely evicted. Further exploration of these possibilities may provide new insights into the ways that *T. gondii* and other disease-causing parasites use vacuoles to protect themselves.
